# Global nitrogen budgets in cereals: A 50-year assessment for maize, rice, and wheat production systems

**DOI:** 10.1038/srep19355

**Published:** 2016-01-18

**Authors:** J. K. Ladha, A. Tirol-Padre, C. K. Reddy, K. G. Cassman, Sudhir Verma, D. S. Powlson, C. van Kessel, Daniel de B. Richter, Debashis Chakraborty, Himanshu Pathak

**Affiliations:** 1 International Rice Research Institute, IRRI-India, NASC Complex, DPS Marg, Pusa, New Delhi 110012, India; 2 University of Nebraska, Department of Agronomy and Horticulture, 234 Whittier Research Building, Lincoln LE 68583-0857, USA; 3 Dr. YS Parmar University of Horticulture & Forestry, Department of Soil Science & Water Management, Nauni, Solan - 173 230, Himachal Pradesh, India; 4 Rothamsted Research, Department of Sustainable Soils & Grassland Systems, Harpenden, Herts, AL5 2JQ, UK; 5 University of California, Davis, Department of Plant Sciences, 1 Shields Avenue, Davis, CA 95616, USA; 6 Nicholas School of Environment, Duke University, Durham, NC 27708, USA; 7 Indian Agricultural Research Institute, Division of Agricultural Physics, Pusa Campus, New Delhi 110012, India; 8 Indian Agricultural Research Institute, Centre for Environment Science and Climate Resilient Agriculture, Pusa Campus, New Delhi, 110012, India

## Abstract

Industrially produced N-fertilizer is essential to the production of cereals that supports current and projected human populations. We constructed a top-down global N budget for maize, rice, and wheat for a 50-year period (1961 to 2010). Cereals harvested a total of 1551 Tg of N, of which 48% was supplied through fertilizer-N and 4% came from net soil depletion. An estimated 48% (737 Tg) of crop N, equal to 29, 38, and 25 kg ha^−1^ yr^−1^ for maize, rice, and wheat, respectively, is contributed by sources other than fertilizer- or soil-N. Non-symbiotic N_2_ fixation appears to be the major source of this N, which is 370 Tg or 24% of total N in the crop, corresponding to 13, 22, and 13 kg ha^−1^ yr^−1^ for maize, rice, and wheat, respectively. Manure (217 Tg or 14%) and atmospheric deposition (96 Tg or 6%) are the other sources of N. Crop residues and seed contribute marginally. Our scaling-down approach to estimate the contribution of non-symbiotic N_2_ fixation is robust because it focuses on global quantities of N in sources and sinks that are easier to estimate, in contrast to estimating N losses per se, because losses are highly soil-, climate-, and crop-specific.

Nitrogen is the *sine qua non* of contemporary high-yield agriculture because it is required in large quantities in plant proteins that help convert solar radiation into carbohydrates that drive plant growth. More than 100 Tg N yr^−1^ of reactive N is produced industrially by the Haber-Bosch process^1^,^2^ using fossil fuels as energy sources. Of this, 50% is applied to three major cereals (maize, 16%; rice, 16%; and wheat, 18%) that provide the bulk of human food calories and proteins consumed either directly as grain or indirectly through livestock products. Without fertilizer-N, global food production would be sufficient for less than half of the current population of 7.3 billion^3^. To produce food for an additional 2 to 3 billion people by 2050, cereal crop production must increase accordingly. Greater production requires more N uptake because cereal seeds contain a large amount of storage protein reserves, and protein contains about 6% N. It is almost certain that additional N fertilizer will be required globally which can be offset to some extent by management practices that improve N use efficiency^4^,^5^,^6^. When adequate N is applied, however, reactive forms of N (ammonia, nitrate, nitrogen oxides) are lost to the environment, causing water pollution, climate forcing, and loss of biodiversity^7^,^8^,^9^,^10^,^11^,^12^.

Although large uncertainties are associated with it, it is estimated that about 67% of fertilizer-N used globally is eventually converted back to non-reactive atmospheric N_2_ and that nearly 33% as reactive N contributes to its transient effects in different ecosystems^7^. More reliable assessments of various sources of N in the environment largely depend on improved estimates of the global N budget in agriculture.

A powerful approach for assessing the fate of N is to construct an N budget at a global scale^13^,^14^,^15^. While recognizing limitations to N budget estimates and the data on which they depend, this scaling-down approach avoids the difficulties associated with scaling-up from location-specific measurements. Location-specific approaches are challenged by large spatial and temporal variability in rates of N harvested by crops and losses to the environment due to the enormous heterogeneity in physical, chemical, and biological properties of soil governing N transformations and transportation^16^. Here, we construct global cumulative N budgets for 1961 to 2010 for the production of maize, rice, and wheat from data on crop production area, yield, and N content of harvested grain and straw, fertilizer-N input, and changes in soil-N reserves (Table S1). The strengths of this scaling-down approach to arrive at the different components of a global N budget for crop production are the (i) close relationship between harvested grain and N content for the major cereals^17^ and (ii) the reporting of relatively accurate crop yields and fertilizer use in global databases. Changes in N reserves in soil, mostly in the soil organic nitrogen (SON), are difficult to estimate because of the lack of a global database. We therefore base these latter estimates on directly measured changes in soil-N in 114 long-term field experiments that are representative of the world’s major cereal production systems. These long-term experiments are located in 21 countries that account for 67% of the global production of the three cereals^18^.

## Results

**Figure 1:**
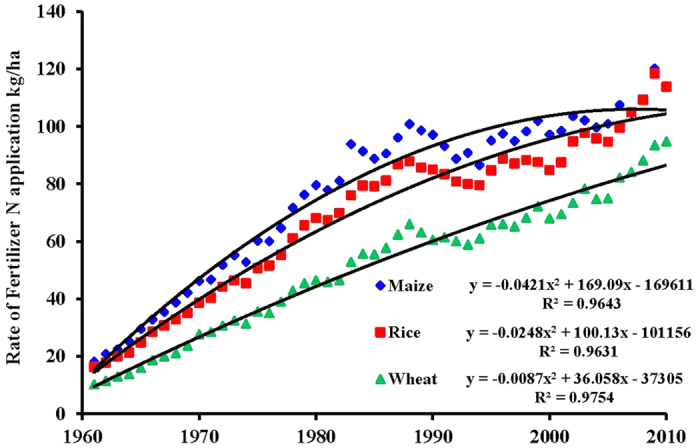
Trends in global averages of fertilizer-N application rates in maize, rice, and wheat.

**Figure 2:**
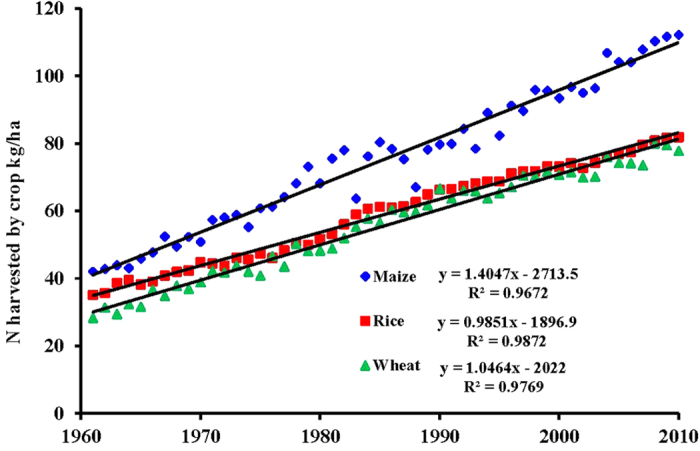
Trends in global averages of total N harvest by maize, rice, and wheat.

Globally during 1961–2010, maize, rice, and wheat received a total of 1594 Tg of fertilizer-N. The N application rate (kg ha^−1^ yr^−1^) showed a curvilinear time trend (Fig. 1), with 50-year averages of 80, 71, and 52 in maize, rice, and wheat, respectively (Table S2). A two-phase trend in N fertilizer rates was used with a faster rate from X to Y until 1980s followed by a slower rate thereafter, which is consistent with actual trends in the USA and Europe^19^. Based on our global assessment, the N harvest (removed from fields in grain and straw) by the three major cereals from 1961 to 2010 was 506 Tg for maize, with a confidence limit (CL) of 491 to 522 Tg; 429 Tg for rice, with a CL of 414 to 444 Tg; and 616 Tg for wheat, with a CL of 586 to 646 Tg, totaling 1551 Tg of N, with a CL of 1514 to 1589 Tg (Table 1). The differences among the three cereals were partly caused by differences in area harvested during 1961 to 2010 (maize 6465 Mha, rice 7138 Mha, and wheat 11051 Mha; Table S2). Unlike the curvilinear time trends of fertilizer-N application, nitrogen harvested by the crop on a per hectare basis showed linear increases from 1961 to 2010, with 50-year averages of 78 kg ha^−1^ for maize, 60 kg ha^−1^ for rice, and 56 kg ha^−1^ for wheat (Table S3 and Fig. 2). Of the total 1551 Tg of crop N harvested by the three cereals, the amount of N derived from applied fertilizer-N (NdF) was 746 Tg, with CL of 717 to 775 Tg (48% of total crop N harvest), and this represented 47% of the 1594 Tg of applied fertilizer-N (Table 1). The remaining 848 Tg of applied N not accounted for by the crop harvest was most likely lost to the environment (Table 2). For the estimations of NdF and fertilizer-N losses, we used globally published data of fertilizer-N recovery (RE_N_; kg fertilizer N taken up kg^−1^ fertilizer N applied) in the three cereals (Table S3)^20^. The RE_N_ of the three cereals was 56% for maize, 36% for rice, and 48% for wheat. Among the cereals, maize had the highest NdF (287 Tg), which was 57% of the crop N harvest. The corresponding percentages for rice and wheat were 43% and 45%, respectively. Expressed on an area basis, NdF values were the highest for maize and the lowest for rice (Table 1).

Based on data from the 114 long-term studies, we estimated that N reserves declined by about 8% in soils growing maize (total of 32 Tg or 5 kg ha^−1^) or wheat (62 Tg or 6 kg ha^−1^) but increased by 4% (26 Tg or 4 kg ha^−1^) under rice (Tables 1 and 2). The magnitude of input of soil N follows general soil quality characteristics of land typically used for these three cereals. For example, average C and N concentrations in the rice soils in the 114 long-term studies were 18 and 2.2 g kg^−1^ (SE ± 1.0 and 0.10), followed by maize (14 and 1.3 g kg^−1^, SE ± 0.4 and 0.04) and wheat (8 and 1.2 g kg^−1^, SE ± 0.8 and 0.09)^18^.

Global N budgeting in the three cereal cropping systems over a 50-year period indicates that 48% of the crop N harvest (equivalent to 737 Tg N; CL 664 to 810 Tg) is derived from sources of N other than fertilizer and soil (Table 1). Rice derived the majority of its N harvest from other sources (63%) whereas maize and wheat were less dependent on these sources (37% and 45%, respectively) (Table 1). On a per hectare annual basis, rice had the greatest amount of N from other sources (38 kg ha^−1^), followed by maize (29 kg ha^−1^) and wheat (25 kg ha^−1^).

**Figure 3:**
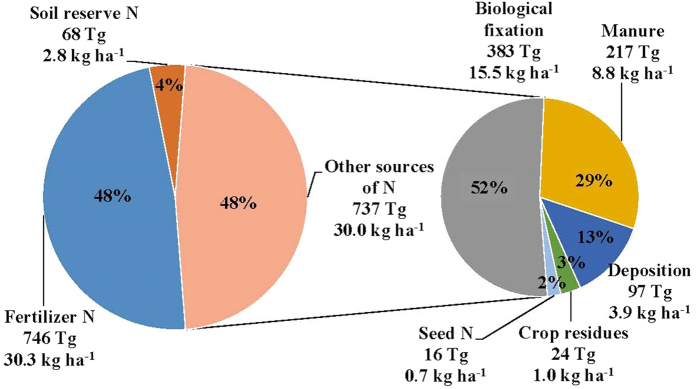
Global estimates of sources of N in crop harvest of maize, rice, and wheat production systems: total (Tg) for 50 years (1961–2010) with percentages and per hectare (kg ha^−1^).

The source of N other than fertilizer and soil reserves would include (1) manure, (2) recycling of N from above-ground crop residues, (3) atmospheric N deposition (both wet and dry), (4) non-symbiotic biological N_2_ fixation (BNF) in soil and plant systems, and (5) seed. Our estimates indicate that, after accounting for the losses, BNF contributed the most, 383 Tg N (15.5 kg ha^−1^), which is 25% of the N harvest of 1551 Tg and 52% of non-fertilizer and non-soil sources of 737 Tg. Manure is the second highest input (217 Tg), followed by deposition (97 Tg), crop residue (24 Tg), and seed (16 Tg) (Fig. 3). Of the total of 578 Tg of manure N applied to the three cereals, 38% (217 Tg) was recovered by the crop and the remaining 62% (361 Tg) was lost to the environment (Table 2). Manure application is a common practice in cereal-based systems in many parts of the world^21^,^22^,^23^,^24^. Manure is often applied on the soil surface and the applications are not accomplished efficiently, thus leading to large losses. Crop residues have many competing uses, including off-site use, and a significant amount is burned on-farm (SI). Based on the available global data on residue recycling and their N availability to crops, we estimated that the N contribution from the return of above-ground crop residue could be about 24 Tg to the total crop N of the three cereals (Table S4 and Fig. 3). Recovery efficiency of N from residue indicates lower than 10% recovery by a crop^25^. Atmospheric deposition contributed nearly 97 Tg to the total crop N by these cereals (Fig. 3). Since the dynamics of N deposited by wet and dry deposition are likely to be similar to the fertilizer-N, fractions of losses were assumed to be similar and were estimated to be 110 Tg by the three cereals (Table 2). Non-symbiotic BNF is difficult to measure because of methodological constraints^26^,^27^ and thus few data are available at a large scale. Accounting for all other N inputs in the present N budgeting allowed relatively more accurate estimations of non-symbiotic BNF in maize, rice, and wheat by the difference method (Methods, Equation S3). The estimates reveal BNF as the largest input of 383 Tg (80% of 478 Tg), with the highest of 160 Tg (80% of 200 Tg) in rice (Table 2). On a per hectare basis, BNF contributed 13, 22, and 13 kg to the N harvest of maize, rice, and wheat, respectively. Since BNF occurs close to the soil and plant, the efficiency of N contribution to the crop was assumed to be 80%.

Our estimates for the 50-year duration (1961–2010) for the three cereals show that the total N input of the three cereals together was 3122 Tg of N, of which 51% was from N fertilizer (Table 2). Of the total of 3122 Tg, BNF and manure accounted for 15% and 19%, respectively, followed by crop residue (8%), deposition (about 7%), and seed (less than 1%). The N output was estimated to be 3190 Tg, of which the crop harvested 48%, equal to 1551 Tg, whereas the remaining 52% or 1639 Tg of N input was lost (Table 2). In addition, soil-N declined by about 68 Tg.

**Figure 4:**
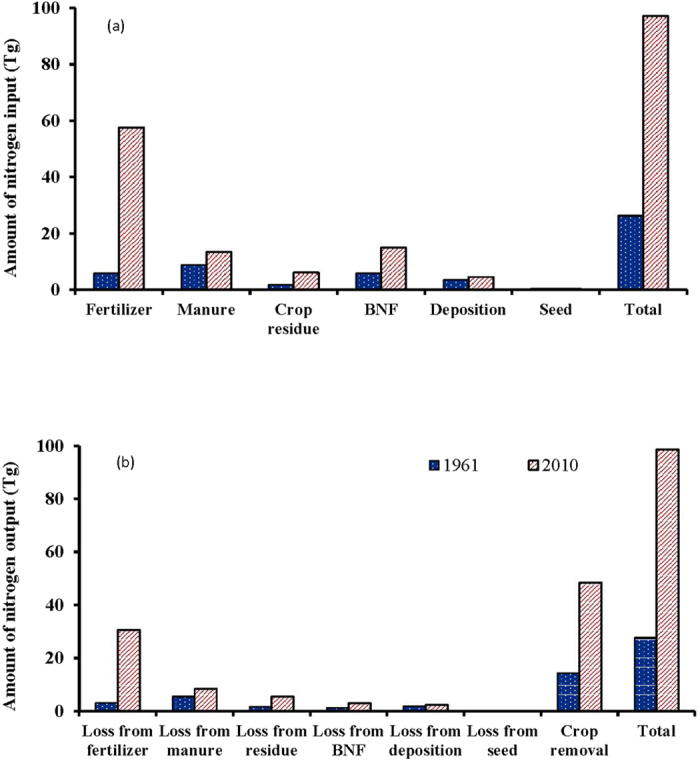
Estimates of N inputs (**a**) and outputs (**b**) in initial (1961) and final (2010) years.

Initial (1961) and final (2010) estimates of total inputs for three cereals show about a 10 times increase in fertilizer-N use from 5.9 Tg in 1961 to 57.6 Tg in 2010 (Fig. 4). Crop residue and non-symbiotic N_2_ fixation increased about 3 times during the same duration but increases in manure N and deposition were less than 50%. Among the outputs, the maximum of 48.5 Tg was through crop removal followed by loss from fertilizer-N of 30.6 Tg. Outputs from the losses of other inputs ranged from 2 to 8 Tg.

## Discussion

This is the first attempt to construct a global N budget for cereals, which is based on N removed by the crop through harvest (grain and above-ground straw). The crop N was accurately determined from a large data set of relatively stable traits of plant N concentrations and the harvest indices of the three cereals available in the literature (Methods and SI). Likewise, the estimates of the fraction of fertilizer-N in the crop (NdF) using the RE_N_ approach were made from a large number of global studies conducted in a wide diversity of maize, rice, and wheat agro-ecosystems. The analysis suggested that an average of 47% of the applied fertilizer-N was recovered by these crops in the year of application. When the soil-N reserve has reached a near steady-state, the remaining N may be considered lost^28^.

An accurate estimate of changes in soil-N resulting from various processes of outputs and inputs and the supply to crops is the most critical to balancing an N budget sheet. We used data on net changes in soil-N (∂N at 30-cm soil depth) from 114 long-term experiments located at 100 sites globally over time scales of decades under a wide range of land management and climate regimes^18^. The small net changes in soil-N during the course of the long-term experiments are consistent with the fact that a near steady-state is typically achieved in fields under a continuous cropping regime^29^. Hence, we assumed that the estimates of changes in soil-N reserve in these long-term studies represent a reasonable proxy of changes in soil-N content in the major cereal production systems worldwide. Typically, between 10% and 40% of the fertilizer-N applied to cereals is assimilated into the soil through microbial biomass and crop residue during the season of application^30^,^31^ and in a near steady-state situation this quantity is roughly balanced by the N released from SON through mineralization. Nevertheless, we accounted for the small changes in soil-N reserve in our N budgeting and therefore provided robust estimates for the three cereals.

Inputs of N through non-symbiotic N_2_ fixation, manure, atmospheric deposition, and from crop residues are beneficial to farmers because they can reduce the necessary applications of fertilizer-N and help maintain soil-N reserves. Because of a lack of data on the contributions of N from sources other than fertilizer and soil to the crop N of three cereals, we suspect that the importance of these contributions to underpin the sustainability of cereal production systems has not been widely appreciated. Often, uncertainties are associated with N budgeting, especially at the global scale. The paucity of data for some components of the global N budget is such an uncertainty. We used large data sets generated during the last several decades, which allowed us to make some assumptions. There may be some uncertainties in our estimates of changes in soil-N because we were not able to consider soil depth below 30 cm. Although data on changes in soil-N in the subsoil (below 30 cm) are limited, no evidence of changes in subsoil-N was found in the Broadbalk Wheat Experiment at Rothamsted after more than 100 years of continuous cropping^32^.

Our estimate shows a relatively larger contribution of N from non-symbiotic or non-legume N_2_ fixation than previously believed. This has been a debatable issue because of the lack of a suitable and direct method to quantify the non-symbiotic N_2_ fixation^27^. It was argued that non-symbiotic N_2_ fixation is a process that occurs on a wide variety of substrates, is nearly ubiquitous in terrestrial ecosystems, and may often represent the dominant pathway for acquiring newly available N^33^. Nonetheless, an indirect approach, developing an N budget, is the only way to estimate the contribution of non-symbiotic N_2_ fixation at a global scale. It could be argued that using a global N budgeting approach that estimates all N inputs and outputs from cereal production systems, except for the non-symbiotic BNF component, incorporates the error from the estimated parameters into the non-symbiotic N_2_ fixation input pool. Although this could be a concern in our budgeting method, there are reasons to believe that this is a robust approach because notably (a) it provides an important complement to other approaches such as those used in the study by Bouwman *et al*.^15^, and (b) it would be highly unlikely if the large number of N budget components that we estimate for the three cereal crops (maize, rice and wheat) were consistently biased in a direction that reduces N inputs and/or increases N outputs resulting in an overestimate of non-symbiotic N_2_ fixation input. Although it is possible to have such large “one-sided” bias, it is much more likely that the biases are random, especially for such a large number of estimated factors. Finally, when the “default” estimates of non-symbiotic N_2_ fixation inputs in our study are compared with those of other reports, they are not beyond the realm of possibility (Table S5). For example, our estimate of non-symbiotic N_2_ fixation for rice is comparable with the value used by Bouwman *et al*.^15^ and much lower than that estimated by Ladha *et al*.^34^ and Bei *et al*.^35^ using two different methods to estimate N_2_ fixation in rice systems. Although our value for wheat is somewhat larger than estimates from some studies (<5–10 kg N ha^−1^)^36^, higher values perhaps up to about 20 kg N ha^−1^ crop^−1^ have been deduced from a long term study with wheat (Table S5). Unfortunately, no non-symbiotic N_2_ fixation estimates from relevant field studies are available for maize but relatively higher inputs of N_2_ fixation in maize may be possible because C4 photosynthetic capability could support endophytic N_2_ fixation^37^. In summary, our study identifies a large, globally significant source of N input to cereal systems not accounted for when tallying up all N inputs (except non-symbiotic N_2_ fixation), all N outputs, and changes in soil-N content. The most likely contributor to this pool of unaccounted for N is non-symbiotic N_2_ fixation, and this finding represents a valuable complement to global N budgets estimated by other approaches. Likewise, by not considering the possible input of N through non-symbiotic N_2_ fixation, an incomplete N budget would be presented as there is ample evidence that there is input of N through non-symbiotic N_2_ fixation in rice and wheat cropping systems from numerous long- and short-term field studies (Table S5).

As no data are available on the efficiency of non-symbiotic fixed N_2_ to be accumulated by the crop, a value of 80% efficiency used has uncertainty. A value lower than 80% will lead to higher inputs of non-symbiotic N_2_ fixation than that estimated in the present study. In addition, the current N budgeting considered only the cereal systems, and symbiotic BNF with legumes as part of the larger cropping system was not considered in the budgeting, as a relatively small percentage of maize-, rice-, and wheat-based cropping systems have legumes as part of their rotation and therefore their contribution of N to the subsequent cereal crop would be limited when considered from a global perspective. In the USA the maize-soybean rotation is important but recent studies document that soil organic matter is either at equilibrium or exhibits a small decline^38^, which means that there is little net N input from BNF. This also follows from the fact that soybean seeds have such a high N concentration that they remove as much or even more N than the input from BNF.

Although fertilizer-N will still remain essential for ensuring the future global food supply, N inputs from other sources appear to be more significant than previously realized. Despite some research efforts, for the most part these inputs have been overlooked for cereal production. Estimating the amounts of N from all sources adequate to meet future demand for food requires assumptions about (1) fertilizer-N use efficiencies, (2) the contribution of N from non-fertilizer sources including non-symbiotic N_2_ fixation, (3) changes in net N input from the soil, and (4) estimates of total N accumulated in the crop. Of these, the first two assumptions will have the largest impact on future fertilizer-N requirements to ensure food security and on the magnitude of environmental impacts associated with fertilizer-N use in agriculture. A better understanding of the magnitude, spatial and temporal variability, and associated N dynamics, of these inputs would certainly enhance efforts to improve the efficiency of fertilizer-N use and ensure global food security. Enhancing non-symbiotic N_2_ fixation in the soil and optimizing use efficiency from all N sources will be especially important if restrictions are placed on the use of fertilizer-N worldwide because of concerns about its contribution to climate change and water pollution. It is also important in regions where low-income farmers cannot afford sufficient quantities of N fertilizer. Moreover, there is an urgent need to accurately quantify the inputs of non-symbiotic biological N_2_ fixation, which appears to be an indispensable source of N for crop production.

## Methods

### N budget

The N budget can be developed by quantifying the inputs and outputs and the change in soil N (∂N = final minus initial) over a period of years^39^. Thus,


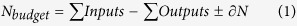


Inputs of N include those from fertilizer (FN), manure (MN), residues of crops (RN), atmospheric deposition (DN), seed (SN), and biological fixation (BNF). Another source of N is lightning, which was estimated to be relatively small and hence was not included as a separate item in the equation: inputs from this source will be included within atmospheric deposition. Outputs include N harvested by the crop (CN) and losses (LN) from various sources through processes such as volatilization, leaching, denitrification, and soil erosion. Thus,


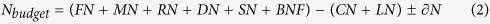


In this equation, all the parameters were estimated except BNF, which is uncertain and difficult to measure. We therefore calculated the contribution of BNF using the following equation:


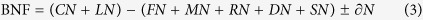


### Estimation of crop (grain + straw) N

Nitrogen harvested by the crop (CN) was calculated as follows:


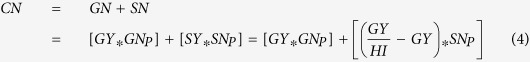


where grain yield (*GY*) is the total of the country-wise grain production of maize, rice, and wheat in kg ha^−1^ over 50 years (1961–2010) obtained from the Food and Agriculture Organization^40^. Percent (g 100 g^−1^) grain N (*GN*_*P*_), % straw N (*SN*_*P*_), and harvest index (*HI*; kg grain biomass kg^−1^ total crop biomass) are averages obtained from a comprehensive inventory of original peer-reviewed publications (Table S1). The use of a single average value (1961–2010) of *HI* for the determination of straw yield (*SY*) of each of maize, rice, and wheat could be debatable because of the apparent claim of improvement over time. However, using three different values (for 1960–70, 1980–90, and 2000–10) yielded similar results for N budgets (data not shown).

### Estimation of fertilizer-N input, crop-N derived from fertilizer, and loss of fertilizer-N

The FAO provides yearly data on N fertilizer inputs in all the global crops^41^. In addition, fertilizer-N inputs in maize, rice, and wheat are reported for more recent years (Table S6)^41^,^42^,^43^,^44^. Average fractions (fertilizer-N input in a cereal to that of total inputs by all the global crops) show 16% each for maize and rice and 18% for wheat, which were used to estimate N inputs by the three cereals during 1961 to 2010. Nitrogen inputs from fertilizer for maize, rice, and wheat for 1995 to 2011 were used to estimate for other years during 1961 to 2010, which may have some errors. However, year-to-year variability in the reported estimates is less than 2%, suggesting a relatively small error in the 50-year N budget (Table S6).

The amount of crop-N derived from fertilizer (NdF) in maize, rice, and wheat was calculated by multiplying fertilizer-N inputs and N recovery efficiency of fertilizer-N (RE_N_) of the respective cereal. The RE_N_ data on the three cereals (55.6%, 36.2%, and 48.3% for maize, rice, and wheat, respectively) were based on a global inventory^20^, which reported values of 65%, 46%, and 57% in maize, rice, and wheat, respectively. Since the reported RE_N_ data came from researchers’ trials conducted globally and RE_N_ would be lower in farmers’ fields^20^, we used 25% lower estimates of RE_N_ in the three cereals. The RE_N_ values were also corrected to include the fraction of N derived from fertilizer in roots and stubbles retained in the soil. To include the fraction of fertilizer-N allocated to roots and stubbles, the RE_N_ values were divided by the following ratios: 0.86 for maize, 0.95 for rice, and 0.87 for wheat. These ratios were calculated using the DeNitrification-DeComposition model (DNDC)^45^. The unrecovered fraction of added N from fertilizer was considered to be lost from the soil.

### Estimation of change (∂N) in soil-N content

The change in soil-N (30-cm soil depth) was calculated as follows:


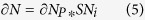


where ∂*N* is the change in soil-N (kg ha^−1^), ∂*N*_*P*_ is the percent (g 100 g^−1^) change in soil-N [(final – initial)*100/initial], and *SN*_*i*_ is initial soil-N (kg ha^−1^) obtained from a recent global inventory of 114 rice-, wheat-, and maize-based LTEs lasting 6–158 years^18^. Most of the collected soil-N data were expressed as g 100 g^−1^. These values were converted to kg ha^−1^, making use of the reported bulk densities from the respective LTEs or, if not reported, from the global inventory of soil bulk densities from different land uses (i.e., lowland, upland, and lowland upland) under temperate, tropical, and subtropical climates. Table S1 shows the total number of data collected for each variable within each subgroup.

### Estimation of the contribution of N through manure, crop residues, deposition, seed, and irrigation water

The FAO provides global data on N inputs through manure to agricultural soils^40^. The contribution of manure-N to maize, rice, and wheat was calculated by extrapolating the FAO data on manure-N use in agricultural soil based on the proportion of fertilizer-N applied to the three major cereals (maize, 16%; rice, 16%; and wheat, 18%) (Table S6). The IPCC^46^ and Smil^13^ assessed the amount of crop residues burned, used for feed, and recycled back to the soil. Accordingly, we calculated the amount of N added to the soil depending on the N contents in residues of these crops (Table S4). In recent years, burning of crop residues is not allowed in many parts of the world and therefore the estimates used are likely to be an underestimation. However, since N contents in residues are relatively low, their effect on the global N budget would be limited. Nitrogen input through atmospheric deposition was calculated by extrapolating the published data^14^ based on area under these crops. Both wet and dry N deposition from the burning of fossil fuels and emissions of N, especially NH_3_ from agriculture, have increased in recent decades^10^. A global average deposition rate (both NOy and NHx) of 3 kg N ha^−1^ yr^−1^ has been estimated, with values reaching up to 50 kg N ha^−1^ yr^−1^ in heavily populated regions of the world^2^,^47^. Although a relatively small fraction of total N deposition occurs on agricultural land^48^, large depositions of reactive N in some areas where major cereals are grown have been reported^47^,^49^. The contributions of seed to maize, rice, and wheat were based on the seed rate of 25, 20, and 80 kg ha^−1^ yr^−1^, respectively, commonly used for these crops and the respective N content in grain. We assumed that globally the contribution of N from irrigation water would be small and did not consider it in the N budgeting; it is recognized that there are certain regions (e.g. in China) where water with a high nitrate concentration is used for irrigation and the irrigation water input is significant. It should be mentioned here that, globally, about 70% of all cereals are rainfed^50^.

### Recovery and loss of N from manure, crop residues, deposition, seed, and BNF

Recovery of N applied through manure was taken as 34–44%^21^,^22^,^23^,^24^ and recovery of crop residue-N was 10%^25^. Since non-symbiotic N_2_ fixation occurs in soil or roots and efficiency of its recovery by the plant is likely to be high, we assumed a recovery of 80%. Likewise, the same logic was used in the case of seed-N; hence, 80% was used. The recovery of N added through deposition was taken as similar to fertilizer-N as both are in the inorganic form and will behave similarly in soil.

### Statistical analysis

The SAS mixed procedure with the Tukey-Kramer test was used to determine the variances and to compare the means for the variables (*GN*_*P*_, *SN*_*P*_, *HI, REN*) among crops^51^. Un-weighted meta-analysis using Meta-Win software^52^ was used to determine the mean and 95% confidence limit (CL) of the primary variables: HI, GNp, and SNp obtained from various studies in the published literature and that were used for calculating GN, SY, SN, and CN from the FAO GY data. The error terms for the secondary variables were determined as follows^53^:

The error terms for the variables *GN* and *SY* were determined using the formula


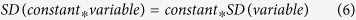

1. The error terms for the variables *SN* and *∂N* were determined using the formula for the SD of the product of two variables:
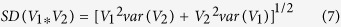
where *V*_1_ and *V*_2_ stand for the two variables and *var* stands for the variance.
2. The error terms for the variables *CN, NdF*, and *X* (CN − NdF + ∂*N*) were determined using the formula for the SD of the sum or difference of two variables:

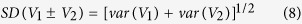


The 95% confidence limit was determined for each parameter as 95% CL = mean ± (1.96 * *sd*).

The 95% CL of the totals (rice [1] + wheat [2] + maize [3]) is given by


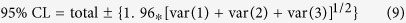


## Additional Information

**How to cite this article**: Ladha, J. K. *et al*. Global nitrogen budgets in cereals: A 50-year assessment for maize, rice, and wheat production systems. *Sci. Rep.* **6**, 19355; doi: 10.1038/srep19355 (2016).

## Supplementary Material

Supplementary Information

## Figures and Tables

**Table 1:** Fifty-year global N budget (Tg, 1961–2010) in maize, rice, and wheat production systems.

Crop	Crop N harvested (CN)	N derived from fertilizer (NdF)	Change in soil N (∂*N*C)	N derived from non-fertilizer and non-soil sources (X = CN − NdF + ∂*N*C)
Maize				
Tg	506	287	−32	187
95% CL	491 to 522	267 to 312	−60 to −5	146 to 223
kg ha^−1^	78.3	44.7	−4.9	28.6
95% CL	65.7 to 80.7	27.7 to 45.0	−9.3 to −0.7	23.3 to 43.2
Rice				
Tg	429	184	26	271
95% CL	414 to 444	175 to 191	−11 to 63	231 to 313
kg ha^−1^	60.1	25.6	3.6	38.1
95% CL	51.9 to 62.2	24.2 to 31.6	−1.6 to 8.9	29.1 to 42.6
Wheat				
Tg	616	275	−62	279
95% CL	586 to 646	251 to 285	−94 to −31	239 to 332
kg ha^−1^	55.7	24.3	−5.6	25.9
95% CL	47.6 to 58.5	22.5 to 31.2	−8.5 to 2.8	16.4 to 28.2
Total				
Tg	1551	746	−68	737
95% CL	1514 to 1589	717 to 775	−125 to −13	664 to 810
kg ha^−1^	62.9	30.0	−2.8	30.2
95% CL	61.4 to 64.5	28.9 to 29.1	−5.1 to −0.6	27.1 to 33.1

CL = Confidence limit.

**Table 2:** Global N budget (Tg, 1961–2010) in maize, rice, and wheat production systems.

Inputs	Maize	Rice	Wheat	Total
Fertilizer	516.9	507.5	569.0	1593.5
Manure	187.5	184.1	206.4	578.1
Crop residue	92.5	65.3	87.1	244.9
Biological fixation	102.7	200.0	175.7	478.4
Deposition	54.3	60.0	92.8	207.1
Seed	2.2	1.6	16.3	20.0
Total	956.2	1018.5	1147.3	3122.0
Outputs				
Crop harvest	506.4	428.7	616.0	1551.1
Loss from fertilizer	229.7	323.7	294.1	847.5
Loss from manure	123.8	103.1	134.2	361.0
Loss from residue	83.3	58.8	78.4	220.4
Loss from biological fixation	20.5	40.0	35.1	95.7
Loss from deposition	24.1	38.2	48.0	110.4
Loss from seed	0.4	0.3	3.3	4.0
Total	988.2	992.8	1209.1	3190.1
Change in soil N	−32.0	25.7	−61.8	−68.1
