# Supplementary material for: Global nitrogen budgets in cereals: A 50-year assessment for maize, rice, and wheat production systems
Source: Sci Rep. 2016 Jan 18;6:19355. doi: 10.1038/srep19355 (PMC4726071; doi:10.1038/srep19355)
Supplement: Supplementary Information [file srep19355-s1.docx]

**Supplementary Information**

**Global nitrogen budgets in cereals: A 50-year assessment for maize, rice, and wheat production systems**

JK Ladha^[[1]](#footnote-1)^, A Tirol-Padre^1^, CK Reddy^1^, KG Cassman^2^, Sudhir Verma^3^, DS Powlson^4^, C van Kessel^5^, Daniel de B. Richter^6^, Debashis Chakraborty^7^, and Himanshu Pathak^8^

^1^ International Rice Research Institute, IRRI-India, NASC Complex, DPS Marg, Pusa, New Delhi 110012, India.

^2^ University of Nebraska, Department of Agronomy and Horticulture, 234 Whittier Research Building, Lincoln LE 68583-0857, USA.

^3^ Dr. YS Parmar University of Horticulture & Forestry, Department of Soil Science & Water Management, Nauni, Solan - 173 230, Himachal Pradesh, India.

^4^ Rothamsted Research, Department of Sustainable Soils & Grassland Systems, Harpenden, Herts, AL5 2JQ, UK.

^5^ University of California, Davis, Department of Plant Sciences, 1 Shields Avenue, Davis, CA 95616, USA.

^6^ Nicholas School of Environment, Duke University, Durham, NC 27708.

^7^ Indian Agricultural Research Institute, Division of Agricultural Physics, Pusa Campus, New Delhi 110012, India

^8^ Indian Agricultural Research Institute, Centre for Environment Science and Climate Resilient Agriculture, Pusa Campus, New Delhi, 110012, India

**Data sources.** The Food and Agriculture Organization’s (FAO) global database is the most comprehensive source of statistics on area, crop production, and yield of maize, rice, and wheat, and N-fertilizer use (see FAOSTAT agricultural data available at http://apps.fao.org). Total global harvested area, grain yield, and N-fertilizer used were summarized for a period of 50 years (1961-2010) and across countries (143-167 countries for maize, 110-118 countries for rice, and 84-125 countries for wheat). Crop harvest index (HI) to estimate straw yield, grain and straw N concentrations to calculate crop N content, recovery efficiency of fertilizer N to calculate NdF, and change in soil N reserve to estimate soil N contribution were based on in-depth literature surveys of peer-reviewed publications using ISI Web of Science (Thomson Reuters) and Google Scholar (Google). Table S1 provides the number of data points used. The change in soil-N (30-cm soil depth) [(final – initial)*100/initial] was obtained from a recent global inventory of 114 rice-, wheat-, and maize-based long-term experiments (LTEs) lasting 6-158 years^1^. A total of 435 observations covering three cereals derived from 21 countries were used for estimating the changes. Most LTEs had replicated treatments comprising a range of agroclimatic zones, soil type, management practices and rates of fertilizer N inputs therefore providing global representation.

**Harvested area, fertilizer-N application, and grain production.** During the 50-year period, wheat had the highest global harvested area of 11051 million ha, followed by rice (7138 million ha) and maize (6465 million ha). Although wheat area was 54.8% to 70.9% higher, grain production was only less than 1% higher than that of maize and rice, resulting in significantly lower average productivity of wheat (2144 kg ha^-1^) than of maize (3624 kg ha^-1^) and rice (3195 kg ha^-1^; paddy or un-milled rice) (Table S2).

**Variation in data points of plant- and soil-N.** Plant and soil-N variables showed significant differences among the three cereals (Table S3). Wheat had the highest grain N concentration (g 100 g^-1^) of 1.84, which was 37.3% higher than that of maize and 62.8% higher than that of rice. Wheat had the lowest N concentration of 0.56 g 100 g^-1^ in straw, with maize showing the highest value (0.80 g 100 g^-1^) and rice (0.69 g 100 g^-1^) an intermediate value. For HI, maize (0.494) and rice (0.481) showed similar values, with a lower value for wheat (0.423).

Changes in soil-N based on the analyses of 114 long-term experiments indicated a significant decline of 8% in soil N in maize and wheat cropping systems and an increase of 4% soil-N in rice systems (Table S3). Rice soil showed an N reserve of 4595 kg ha^-1^, which was 36% and 40% higher than that of wheat and maize cropping systems, respectively.

**Contribution of N from crop residue to the total crop N harvest of maize, rice, and wheat.** Globally, about 80.74 Pg (petagrams or 80737 Tg) of crop residue or stubble biomass (leaf and stem) were estimated to be produced by maize, rice, and wheat in 50 years (Table S4). The developing world contributed nearly 40% and the developed world the remaining 60% for maize and wheat residue, and about 95% vs 5% by the developing and developed countries, respectively, for rice residue. Residues have many competing uses and, in the past, the majority of cereal residues were either routinely removed from the field or burned^2^. However, with the progressive adoption of conservation agriculture and minimum tillage practices around the world over the past two decades, residue retention is becoming increasingly common, particularly in wheat production systems^3^. Consequently, in the preparation of global N budgets, it is necessary to account for N recycled in cereal residues, and the contributions of N recycled from crop residues becoming available for subsequent crop N uptake, largely a relatively recent phenomenon within the context of the 50-year time frame used in this study. Unfortunately, there are few determinations of the fate of cereal residue-N following grain harvest. Smil^2^ estimated that 35% of crop residues (CR) were burned in developing countries and 15% in developed countries, whereas the IPCC estimates^4^ were 25% and 10%, respectively. Using these two estimates, and assuming that all un-burned residues and those not used for feed were recycled on-site, a maximum of 37 Pg of residue (equivalent to 245 Tg of N) out of 81 Pg of cereal residue generated globally over the past 50 years could potentially have been recycled back into the soil (Table S4). Data on the recovery of crop residue N in maize, rice, and wheat are scarce. Based on a ^15^N study carried out in nine countries, less than 10% of total residue N was recovered by the first crop and a negligible amount by the succeeding crops^5^. This would imply that 25 Tg of N would be the upper limit of residue N that is recovered by the three cereals, which represents about 3.4% of 737 Tg of N coming from sources other than synthetic fertilizer-N and soil-N.

**Contribution of N from non-symbiotic biological nitrogen fixation.** Table S5 shows a quantification of non-symbiotic N_2_ fixation in rice and wheat at the global scale. Except for a study that used the direct method (^15^N feeding) of measuring BNF^6^, all the other studies were based on the N balance method. Eight out of 11 published studies were in rice systems and the remaining in wheat systems. The amount of BNF in rice was in the range of 18 to 51 kg ha^-1^ and in wheat from 20 to 35 kg ha^-1^ (Table S5). However in case of wheat, a significant amount of deposition was measured at Rothamsted Experimental Station which led to a lower estimate of BNF of 10 to 20 kg ha^-1^ (David Powlson, personal communication).

**References**

1. Ladha, J. K., Reddy, C. K., T-Padre, A. & Kessel, C. V. Role of nitrogen fertilization in sustaining organic matter in cultivated soils. *J. Environ. Qual.* **40**, 1756-1766 (2011).
2. Smil, V. Nitrogen in crop production: an account of global flows. *Global Biogeochem. Cycles.* **13,** 647-662 (1999).
3. Friedrich, T., Derspsch, R. & Kassam, A. Overview of the global spread of conservation agriculture. Field Actions Science Reports. Special Issue 6. (2012) Available at: <http://factsreports.revues.org/1941>. (Accessed: 6 November 2012).
4. IPCC. Guidelines for National Greenhouse Gas Inventories (Revised). (1996) Available at: http://[www.ipcc-nggip.iges.or.jp/public/gl/invs6c](http://www.ipcc-nggip.iges.or.jp/public/gl/invs6c). (Accessed: March, 2014).
5. IAEA. Management of crop residues for sustainable crop production. IAEA-TECDOC-1354 [243] (IAEA, Vienna, Austria, 2003).
6. Bei Qicheng, *et al.* Heterotrophic and phototrophic ^15^N_2_ fixation and distribution of fixed ^15^N in a flooded rice-soil system. *Soil Biol. Biochem.* **59,** 25-31 (2013).
7. FAO (Food and Agriculture Organization of the United Nations). FAO statistical databases. (2015) Available at: <http://faostat.fao.org>. (Accessed: April, 2015).
8. Ladha, J. K., Pathak, H. P., Krupnik, T. J., Six, J. & Kessel, C. van. Efficiency of fertilizer nitrogen in cereal production: retrospect and prospect. *Adv. Agron.* **87,** 85-156 (2005).
9. Ladha, J. K. *et al.* Long-term effects of urea and green manure on rice yields and nitrogen balance. *Soil Sci. Soc. Am. J.* **64,** 1993-2001 (2000).
10. Pampolino, M. R., Laureles, E. V., Gines, H. C. & Buresh, R. J. Soil carbon and nitrogen changes in long-term continuous lowland rice cropping. *Soil Sci. Soc. Am. J.* **72,** 798-807 (2008).
11. App, A. *et al.* Estimation of the nitrogen balance for irrigated rice and the contribution of phototrophic nitrogen fixation. *Field Crops Res.* **9**, 17-27 (1984).
12. Jenkinson, D. S. Organic matter and nitrogen in soils of the Rothamsted classical experiments. *J. Sci. Food Agric.* **24,** 1149-1150 (1993).
13. Powlson, D. S., Pruden, G., Johnson, A. E. & Jenkinson, D. S. The nitrogen cycle in the Broadbalk Wheat Experiment: recovery and losses of ^15^N-labelled fertilizer applied in spring and inputs of nitrogen from the atmosphere. *J. Agric. Sci. Camb.* **107,** 591-609 (1986).
14. Gupta, V. V. S. R., Roper, M. M. & Roger, D. K. Potential for non-symbiotic N_2_-fixation in different agroecological zones of southern Australia. *Aust. J. Soil Res.* **44,** 343-354 (2006).
15. FAO. Fertilizer requirements in 2015 and 2030. [1-29] (FAO, Italy, 2000).
16. Bruinsma, J. World agriculture: towards 2015/2030: *An FAO perspective. Economic and social development department,* [Bruinsma, J. (ed.)] [432] (Food and Agriculture Organization, Earthscan publications Ltd., London, UK, 2003).
17. Heffer, P. Assessment of Fertilizer Use by Crop at the Global Level 2006/07 – 2007/08. [1-12] (International fertilizer industry association, France, 2009).
18. Heffer, P. Assessment of Fertilizer Use by Crop at the Global Level 2010-2010/11. [1-9] (International Fertilizer Industry Association, France, 2013).

Table S1 | Source and details of primary and secondary data sets

| Parameter | Source | Number of observations used for the estimations | | |
| --- | --- | --- | --- | --- |
|  |  | Maize | Rice | Wheat |
| Crop harvested area (m ha)  Grain yield (kg ha^-1^ or Tg)  Fertilizer N use (kg ha^-1^ or Tg) | (7) | 50  (143-167)* | 50  (110-118)* | 50  (84-125)* |
| Harvest index (ratio) | Published literature | 2512 | 2503 | 1074 |
| Grain N (g 100 g^-1^) | Published literature | 2433 | 2258 | 891 |
| Straw N (g 100 g^-1^) | Published literature | 2325 | 2255 | 663 |
| Recovery efficiency of fertilizer N - RE_N_ (kg ^1^N recovered kg-^1^N applied) | (8) | 48 | 149 | 356 |
| Change in soil N reserve (g 100 g ^-1^) | (1) | 136 | 41 | 258 |

* Number of countries globally

Table S2 | Harvested area, fertilizer-N application, and grain production used to construct the global N budget in cereals

| Variable | Maize | Rice | Wheat |
| --- | --- | --- | --- |
| Total harvested area (million ha) 1961-2010 (sum of 50 years) | 6465 | 7138 | 11051 |
| Total N application (Tg) 1961-2010 (sum of 50 years) | 517 | 508 | 569 |
| Average N application (kg ha^‑1^ yr^-1^)  95% CL | 80.0  72.7 to 89.3 | 71.1  59.0 to 73.7 | 51.5  46.5 to 59.6 |
| Grain production (Tg) 1961-2010 (sum of 50 years) | 23428 | 22809 | 23689 |
| Grain yield average (kg ha^‑1^ yr^-1^)  95% CL | 3624  3217 to 3764 | 3195  2922 to 3359 | 2144  1973 to 2311 |

Table S3 | Statistics of plant and soil data on maize, rice, and wheat for constructing the global N budget in cereals

| Variable | Maize | Rice | Wheat |
| --- | --- | --- | --- |
| Harvest index (ratio) | 0.494 a^1^ | 0.481 b | 0.423 c |
| 95% CL | 0.455 to 0.533 | 0.442 to 0.520 | 0.363 to 0.482 |
| Grain N (g 100 g^-1^) | 1.34 b | 1.13 c | 1.84 c |
| 95% CL | 1.30 to 1.38 | 1.09 to 1.18 | 1.77 to 1.90 |
| Straw N (g 100 g^-1^) | 0.80 a | 0.69 b | 0.56 c |
| 95% CL | 0.76 to 0.84 | 0.65 to 0.73 | 0.48 to 0.64 |
| Crop uptake (kg ha^-1^)^2^ | 78.32 a | 60.06 b | 55.74 b |
| 95% CL | 65.68 to 80.65 | 51.89 to 62.15 | 47.58 to 58.45 |
| Recovery efficiency of fertilizer N  - RE_N_ (kg ^1^ N kg^-1^ N) | 0.56 | 0.36 | 0.48 |
| 95% CL | 0.50 to 0.59 | 0.35 to 0.38 | 0.43 to 0.61 |
| Recovery efficiency of manure N - RE_N_ (kg ^1^ N recovered kg-^1^ N applied) | 0.34 | 0.44 | 0.35 |
| Change in soil N (g 100 g^-1^) | -7.62 b | 4.01 a | -8.37 b |
| 95% CL | -14.11 to -1.12 | -1.63 to 9.64 | -12.56 to -4.17 |
| Soil N (kg ha^-1^)^2^ | 3290 b | 4595 a | 3375 b |
| 95% CL | 2918 to 3662 | 3713 to 5478 | 3102 to 3648 |

^1^ In a row, means followed by a common letter are not significantly different from each other at the 5% level by the Tukey pairwise mean comparison.

^2^ Averages of 50 years.

Table S4 | Fifty-year global estimates of crop residue biomass and N (above-ground: stem + leaf) recycled and recovered by maize, rice, and wheat

| Crop | Produced | Recycled | N content | N recovered by crop |
| --- | --- | --- | --- | --- |
|  |  | Tg |  |  |
| Maize | 23790 | 11467 | 92.5 | 9.2 |
| Rice | 24585 | 9441 | 65.3 | 6.5 |
| Wheat | 32362 | 15598 | 87.1 | 8.7 |
| **Total** | **80737** | 36506 | 244.9 | 24.5 |

^_______________________________________________________________________________________________________________^

Based on Smil^2^ and IPCC^4^, we used 30% residue burning and 33% for animal feed plus bedding in developing countries; and 12.5% residue burning and 22.5% for animal feed plus bedding in developed countries. Thus, 37% and 65% of residues were recycled in the developing and developed countries, respectively. The amount of residues generated in developing and developed countries were apportioned based on the area under the three cereals in the respective countries.

Table S5 | Estimates of non-symbiotic/free-living nitrogen fixation in rice and wheat

| S. no. | Location | Annual crop rotation | Crop | Number of crops | Year | Method | Amount (kg ha^-1^ crop^-1^) | Remark | Source |
| --- | --- | --- | --- | --- | --- | --- | --- | --- | --- |
|  | Jiangdu, China | Rice-wheat | Rice | 1 | 2010 | ^15^N feeding | 45 | Most direct method (70 d incubation) | (6) |
|  | Los Baños, Philippines | Rice-rice | Rice | 27 | 1985-98 | N balance | 46 | Excluding deposition | (9) |
|  | Bocol, Philippines | Rice-rice | Rice | 30 | 1968-83 | N balance | 35 | Excluding deposition | (10) |
|  | Los Baños, Philippines | Rice-rice | Rice | 30 | 1964-79 | N balance | 18 | Excluding deposition | (10) |
|  | Maligaya, Philippines | Rice-rice | Rice | 30 | 1968-83 | N balance | 44 | Excluding deposition | (10) |
|  | Los Baños, Philippines | Rice-rice-rice | Rice | 45 | 1963-83 | N balance | 27 | Excluding deposition | (11) |
|  | Los Baños, Philippines | Rice-rice | Rice | 24 | 1966-78 | N balance | 51 | Excluding deposition | (11) |
|  | Maligaya, Philippines | Rice-rice | Rice | 17 | 1968-1976 | N balance | 39 | Excluding deposition | (11) |
|  | Rothamsted, UK | Wheat | Wheat | 115 | 1852-1967 | N balance | 25-35 | Excluding deposition and seed | (12) |
|  | Rothamsted, UK | Wheat | Wheat | 4 | 1979-83 | N balance | 25 | Excluding deposition and seed | (13) |
|  | Avon, Australia | Wheat | Wheat | 17 | 1979-96 | N balance | 20 | Including deposition and seed | (14) |

Table S6 | Estimates of the percentage of world N-fertilizer use for maize, rice, and wheat production

| Years of estimates | Maize | Rice | Wheat | Total | Source |
| --- | --- | --- | --- | --- | --- |
| 1995-97 | 15.2 | 16.8 | 19.6 | 51.6 | (15) |
| 1997-99 | 16.3 | 17.3 | 18.4 | 54.0 | (16) |
| 2006-07 | 17.3 | 15.8 | 17.3 | 50.4 | (17) |
| 2007-08 | 16.8 | 15.6 | 17.3 | 49.7 | (17) |
| 2010-11 | 16.8 | 15.4 | 18.1 | 50.3 | (18) |
| **Average** | **16.48** | **16.18** | **18.14** | **50.8** |  |

1. Correspondence and requests for materials should be address to J.K. Ladha ([j.k.ladha@irri.org](mailto:j.k.ladha@irri.org)); Tel.: +91-11-6676 3000; Fax: +91-11-2584 1801. [↑](#footnote-ref-1)
